# Umbilical acupuncture for insomnia: A protocol for systematic review and meta-analysis

**DOI:** 10.1097/MD.0000000000028037

**Published:** 2021-12-03

**Authors:** Zhi-tao Feng, Da-shi Ying, Zhan-shuang Qiu, Tie Li, Xiao-ru Xu, Ji-yu Yang, Zhi-hong Wang

**Affiliations:** Changchun University of Traditional Chinese Medicine, College of Acupuncture and Tuina, P.R. China.

**Keywords:** insomnia, meta-analysis, protocol, systematic review, umbilical acupuncture

## Abstract

**Background:**

Insomnia is characterized by high incidence, easy recurrence, and difficulty in curing. Serious insomnia not only seriously affects the body organ function but also causes great damage psychological.

Umbilical acupuncture (UA) has fewer side effects and is increasingly used to treat insomnia. This study aimed to systematically review the effectiveness and safety of UA in the treatment of insomnia.

**Methods:**

Literature on UA for insomnia in PubMed, Excerpt Medica Database, the Cochrane Central Register of Controlled Trials, Web of Science, China National Knowledge Infrastructure Database, China Biomedical Literature Database, Chinese Scientific Journal Database, and Wan Fang Database were searched from the creation of these databases to October 3, 2021. In addition, the reference lists of studies meeting the inclusion criteria will also be searched to achieve a comprehensive retrieval of the maximum. All randomized controlled trials of UA for treating insomnia were included. Two reviewers will conduct literature screening, data extraction, and quality evaluation respectively. The main outcome was the Pittsburgh Sleep Quality Index, and the secondary outcomes included clinical efficacy, and safety. RevMan 5.4.1 software was used for mate analysis.

**Results:**

This study aimed to evaluate the current status of UA treatment for insomnia, with the aim of illustrating the effectiveness and safety of UA.

**Conclusion:**

This study will provides a high-quality evidence to evaluate the effectiveness and safety of UA in treating insomnia.

**Registration:**

PROSPERO CRD42021283036.

## 1 Introduction

### 1.1 Description of the condition

Insomnia is a subjective symptom of poor sleep quality and low sleep duration due to personal reasons such as inability to fall asleep or easy to wake up and difficulty in falling asleep.^[1]^ About 30% to 35% of the world's population suffers from some degree of sleep disorder,^[2]^ and according to an epidemiological survey,^[3]^ about 45.5% of the population in China have different levels of sleep problems. In order to continue to sleep, time is too short and difficulty falling asleep once awake was listed as the main symptom. About 50% of the patients show symptoms of 2 or more at the same time, which seriously influences the patient's emotional, and social functions and quality of life. A longitudinal study found that individuals who reported severe insomnia symptoms had a remission rate of only 56% over 10 years.^[4]^ Long-term sleep deprivation can lead to daytime dysfunction, inability to recover physical strength, low work efficiency, poor academic performance, and severe insomnia can increase the risk of coronary heart disease, acute coronary syndrome, anxiety, depression and other diseases.^[5,6]^ At present, Western medicine is the mainstay of treatment for insomnia, and Western drugs mainly include benzodiazepines, nonbenzodiazepines, and melatonin, etc. Benzodiazepines have obvious side effects, including drug resistance and rebound insomnia, and long-term use of elderly patients increases the risk of dementia^[7]^; nonbenzodiazepines can cause taste disorders, headache, dizziness, and other discomfort^[8]^; melatonin has adverse reactions such as dizziness, taste disturbance, and drowsiness.^[9]^ Based on the side effects of drug therapy, medical practitioners are forced to explore better complementary and alternative therapies.^[10]^

### 1.2 Description of the intervention

Umbilical acupuncture (UA) is an emerging therapy established by Professor Qi Yong, which is different from traditional acupuncture.^[11]^ Its theoretical basis is completely rooted in traditional Chinese culture. Due to its precise clinical efficacy, umbilical needle has been widely inherited and spread, and has been accepted and recognized by the international acupuncture community.^[12]^ UA is different from traditional acupuncture, which is a therapeutic method to treat diseases by acupuncture at the umbilical point in a certain way based on the theory of bioholography.^[13]^ UA therapy^[14]^ were included 3 areas, namely, the umbilical wall, the umbilical core, and the umbilical valley. The umbilical wall is the mural tissue surrounding the umbilical hole, and the umbilical core is the scar tissue protruding from the center of the umbilical, and the umbilical valley is located in the skin depression between the 2. Due to the particularity of umbilical anatomy, the umbilical wall is most commonly used in clinical practice. UA is widely used in clinic for its simple operation, low cost, quick curative effect, safety and no side effects.^[15]^ At present, UA is widely used in the treatment of internal, external, women, children, facial diseases and other diseases and sub-health conditioning, the range of treatment of hundreds of diseases.^[16]^ Modern literature research shows that^[17]^ UA is the most widely used in the treatment of insomnia in the treatment of internal diseases.

### 1.3 How the intervention might work

Traditional Chinese medicine (TCM)^[18]^ holds the view that the underlying pathogenesis for insomnia is an imbalance of Yin and Yang, and it believes that the disease location is mainly in the heart, closely related to the liver, spleen and kidney. In TCM theory, umbilical cord, as the center of the whole body vein, connects the viscera and viscera through the whole body veins to communicate inside and outside the human body. It is the place where qi converges and is the most important part of the body. Acupuncture of the umbilical cord can adjust the whole body qi and blood, the purpose is to adjust the balance of Yin and Yang. Umbilical cord is the only way we can connect to the mother from birth, and it is considered the most advanced prenatal diagnosis to obtain fetal health information through umbilical cord blood puncture.^[19]^ Western medicine^[20]^ believes that it is the brain–gut axis, which is the theoretical basis of UA for the treatment of insomnia. The brain–gut axis refers to a bidirectional regulatory system of interactions between the gastrointestinal tract and the brain.^[21]^ UA stimulates the umbilical tract and improves sleep quality by regulating the secretion of sleep-related brain–gut peptide content through the brain–gut axis.^[22]^

### 1.4 Why it is important to do this review

UA treatment of insomnia has the characteristics of simple operation, nontoxic side effects, and positive curative effect, and high safety, and UA has now been extensively applied to the clinical treatment for insomnia in China. However, no study has evaluated the safety and efficacy of UA in the treatment of insomnia in a systematic way. The aim of this study was to systematically evaluate the effectiveness and safety of UA for insomnia so that reliable clinical evidence can be provided.

### 1.5 Objectives

To systematically evaluate the effectiveness and safety of UA for insomnia.

## 2 Methods

### 2.1 Study registration

The protocol was registered on PROSPERO (http://www.crd.york.ac.uk/PROSPERO) and its registration number was CRD42021283036. We will complete this protocol according to the preferred reporting items for systematic reviews and meta-analysis protocols.^[23]^ The changes are described in our full review if needed.

### 2.2 Inclusion criteria for study selection

#### 2.2.1 Types of studies

We will only need randomized clinical trials (RCTs) on UA for insomnia, regardless of whether the blind method and allocation concealment are used.

#### 2.2.2 Types of participants

Participants diagnosed with insomnia were included. Diagnosis criteria include the Diagnostic and Statistical Manual of Mental Disorders (DSM-V, DSM-5),^[24,25]^ International Classification of Sleep Disorders-3,^[26]^ International Statistical Classification of Diseases and Health-Related Problems-10,^[27]^ and Chinese Classification of Mental Disorders-3.^[28]^ No restrictions will be applied to age, gender, ethnicity, or source of cases.

#### 2.2.3. Types of interventions and comparisons

2.2.3

The experimental group was treated with UA; the control group was treated with other therapies, such as Western medicine, acupuncture, moxibustion, auricular needle, and other conventional therapies. The following comparisons were made.

1. UA with Chinese herbal medicine;
2. UA compared with western medicine;
3. UA compared with placebo treatment;
4. UA compared with acupuncture alone;
5. UA compared with moxibustion alone.

If the 2 groups received the same additional active therapy on the basis of the control treatment, the study can also be included.

#### 2.2.4 Types of outcome measures

##### 2.2.4.1 Primary outcomes

Sleep quality will be evaluated using the Pittsburgh Sleep Quality Index^[29]^ as the primary outcome. Improvement in insomnia can be measured by the clinical efficacy and clinical cure rate. Outcomes can be measured simply at the end of treatment.

Possible primary outcomes included the following:

1. Improvement in overall symptoms of insomnia
2. Improvement in quality of life
3. Clinical efficacy or clinical cure rate

##### 2.2.4.2 Secondary outcomes

Secondary outcomes will include: the total scores of the Insomnia Severity Index^[30]^; syndrome according to standards for assessing TCM^[31]^; and adverse events, such as nausea, dizziness, vomiting, and fatigue.

### 2.3 Search methods for identification of studies

#### 2.3.1 Electronic searches

Two independent reviewers (QZS and WJJ) will search the following 8 databases from the inception to October 2021, including China National Knowledge Infrastructure Database, Chinese Scientific Journal Database, China Biomedical Literature Database, Wan Fang Data Chinese Database, PubMed, Cochrane Central Register of Controlled Trials, Web of Science, and Excerpt Medica Database. The combined method of MeSH term and free words was used for literature retrieval. There were no restrictions on language and publication status. The search strategy for PubMed is shown in Table 1. The search strategies of the other databases were established similarly.

**Table 1:** The search strategy for PubMed database.

Number	Search terms
#1	Insomnia [MeSH terms]
#2	Sleeplessness [MeSH terms]
#3	Sleep disorder [MeSH terms]
#4	Dyssomnia [MeSH terms]
#5	#1 or #2 or #3 or #4
#6	Insomnia [Title/abstract]
#7	Sleeplessness [Title/abstract]
#8	Sleep disorder [Title/abstract]
#9	Dyssomnia [Title/abstract]
#10	#6 or #7 or #8 or #9
#11	#5 or #10
#12	Umbilical acupuncture [MeSH terms]
#13	Umbilical needle [MeSH terms]
#14	Umbilical electroacupuncture [MeSH terms]
#15	#12 or #13 or #14
#16	Umbilical acupuncture [Title/abstract]
#17	Umbilical needle [Title/abstract]
#18	Umbilical electroacupuncture [Title/abstract]
#19	#16 or #17 or #18
#20	#15 or #19
#21	Clinical trail [MeSH terms]
#22	Randomized clinical trail [MeSH terms]
#23	Randomized controlled trail [MeSH terms]
#24	#21 or #22 or #23
#25	Randomized clinical trail [Title/abstract]
#26	Randomized controlled trail [Title/abstract]
#27	RCT [Title/abstract]
#28	Clinical trail [Title/abstract]
#29	Random∗ [Title/abstract]
#30	Clinical trail [Publication Type]
#31	#24 or #25 or #26 or #27 or #28 or #29 or #30
#32	#24 or #31
#33	#11 and #20 and #32

#### 2.3.2 Searching other resources

To augment the results of the database search, the bibliographies of the identified studies, relevant reports, and reviews will be manually searched. We will also contact the relevant experts and organizations for information about unpublished and ongoing studies.

### 2.4 Data collection and analysis

#### 2.4.1 Selection of studies

**Figure 1:**
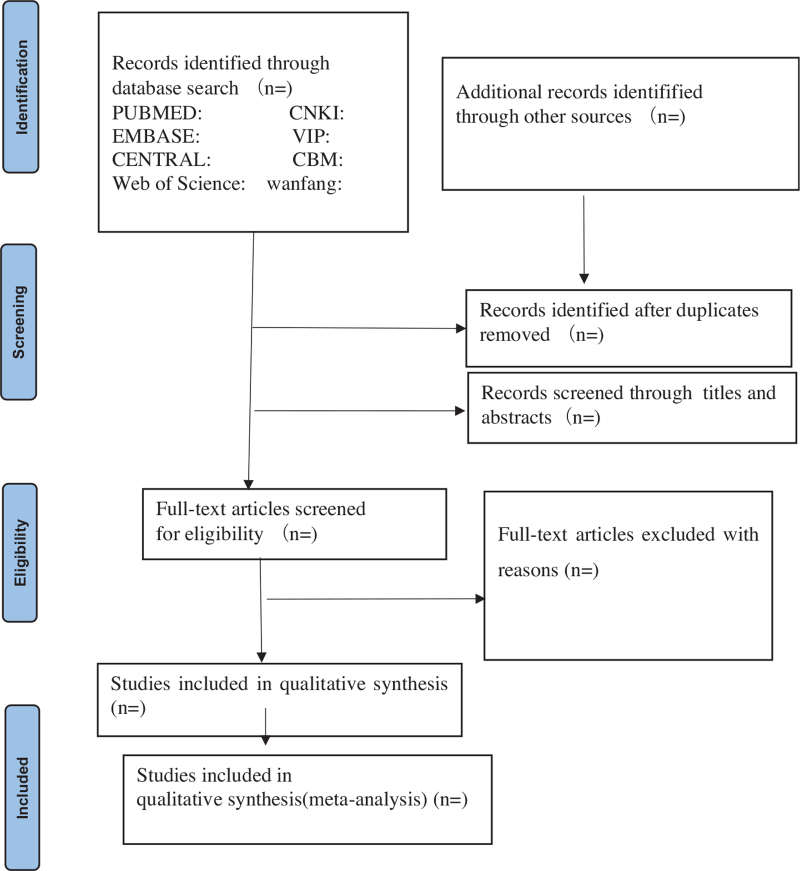
Flow chart of the search process.

We will first use Note Express software (V.3.2) to remove duplicates, and then screen the retrieved studies separately by 2 reviewers (ZLL and QZS) according to the inclusion criteria. Two reviewers (ZLL and QZS) will exclude the papers that do not meet the inclusion criteria by reading the titles and abstracts. Then, the reviewers will check the full texts to determine the final decision according to the criteria. All the screening processes were conducted independently. If the articles information is insufficient, we will try to contact the authors to obtain the necessary details. When 2 reviewers have different opinions, the final decision will be made by the third reviewer (LT). The selection flow process of is shown in the preferred reporting items for systematic reviews and meta-analysis flow chart (Fig. 1).

#### 2.4.2 Data extraction and management

First we design an extraction form that meets the purpose of this system review, which will include the following information from the included studies: participant characteristics, interventions, outcomes, and adverse events. Two investigators (QZS and ZLL) independently completed the data collection form for all eligible studies. The corresponding authors will be contacted to request insufficient or missing information. Disagreements will be resolve by discussion or by appealing to a third author (WZH). The data were stored in Microsoft Excel.

#### 2.4.3 Assessment of risk of bias in included studies

We used the Cochrane risk assessment tool to assess the risk of bias.^[32]^ The methodological quality of RCTs will be independently evaluated by 2 reviewers (FZT and QZS). The following 7 items will be included: random sequence generation, allocation concealment, blinding of participants and caregivers, blinding of outcome evaluator, incomplete outcome data, selective reporting, and other bias. High, low, and unclear assessments were performed for each item. Any disagreement between the 2 reviewers (FZT and QZS) will be resolved by a discussion. Further disagreements were arbitrated by the third author (WZH).

#### 2.4.4 Measures of treatment effect

We will use the mean difference or standard mean difference with 95% confidence intervals as the effect measure for continuous data. Dichotomous outcomes will be analyzed by the risk ratio with 95% confidence interval.

#### 2.4.5 Dealing with missing data

When there are events in the reports that are unclear or do not report data, we will contact the author by phone or email to obtain complete information.

#### 2.4.6 Assessment of heterogeneity and data synthesis

##### 2.4.6.1 Assessment of heterogeneity

We will use RevMan 5.4.1 software to detect the heterogeneity between studies.^[33]^ When *P* < .01, I^2^ > 50%, there is significant heterogeneity between studies; otherwise, heterogeneity is acceptable.

##### 2.4.6.2 Data synthesis

RevMan 5.4.1 will be used for all statistical analyses. We used the random effects model to merge the data. The results of the meta-analyses are presented as forest plots. When the results are unsuitable for combination due to the clinical or methodological heterogeneity, we will perform a descriptive analysis.

#### 2.4.7 Sensitivity analysis

If the result shows high heterogeneity (the I^2^ test is >50%), we will conduct a sensitivity analysis. We will then acquire a stable result of our study.

#### 2.4.8 Subgroup analysis

If there are adequate studies and available data, we will conduct subgroup analysis for different syndrome types of insomnia to explain the heterogeneity among studies.

#### 2.4.9 Assessment of reporting biases

Funnel plots were used to explore the publication bias when 10 or more trials were included.

#### 2.4.10 Grading the quality of evidence

The certainty of a body of evidence will be assessed by using the approach developed by the Grades of Recommendation, Assessment, Development and Evaluation Working Group,^[34]^ involving risk of bias, heterogeneity, indirectness, imprecision, publication bias, and other domains. The certainty level will be rated as high, moderate, low, or very low, and the strength of evidence recommendation will be judged as strong or weak.

### 2.5 Ethics and dissemination

In this study, no individual data from participants were involved, so ethics approval was not required. This systematic review will be published in a peer-reviewed journal.

## 3 Discussion

Insomnia is most common in the elderly, women, mental workers, and people without social backgrounds and has a serious impact on health and quality of life.^[35]^ Due to obvious side effects of drug treatment for insomnia and the high price of cognitive behavioral therapy for insomnia, heavy economic burden for patients and high requirements for doctors, it is not widely used in clinical practice at domestic and overseas.UA for insomnia, has the advantages of quick effect, safety, low price, definite curative effect, and no side effects. UA is widely used in clinical treatment of insomnia in China. Therefore, it is necessary to conduct a systematic review to establish convincing evidence to prove the effectiveness and safety of UA for insomnia. Due to the uneven quality of the literature, such as, few outcome indicators, long publication time, and lack of evidence quality evaluation, the results may be uncertain. Therefore, we will adopt a more rigorous method for systematic review method. We hope that this evidence can help clinicians and health policymakers make clinical decisions on insomnia, and bring good news to patients. However, there may be some potential limitations to this systematic evaluation. First, due to the different types of insomnia, heterogeneity may be greater. Second, the quality of RCTs may be low and there is a risk of bias.

## Author contributions

**Conceptualization:** Zhi-tao Feng, Zhi-hong Wang.

**Data curation:** Da-shi Ying, Zhan-shuang Qiu, Zhi-hong Wang.

**Investigation:** Da-shi Ying, Xiao-ru Xu.

**Methodology:** Da-shi Ying, Tie Li, Ji-yu Yang.

**Project administration:** Zhi-hong Wang.

**Resources:** Zhi-tao Feng.

**Software:** Zhi-tao Feng, Da-shi Ying, Zhan-shuang Qiu, Tie Li, Xiao-ru Xu, Ji-yu Yang.

**Writing – original draft:** Zhi-tao Feng, Zhan-shuang Qiu, Tie Li, Zhi-hong Wang.

**Writing – review & editing:** Zhi-tao Feng, Zhi-hong Wang.

## References

[1] Bastien CH. Insomnia: neurophysiological and neuropsychological approaches. Neuropsychol Rev 2011;21:22–40. 21249453 10.1007/s11065-011-9160-3

[2] Morin CM Drake CL Harvey AG. Insomnia disorder. Nat Rev Dis Primers 2015;1:15026. 27189779 10.1038/nrdp.2015.26

[3] Ye ZJ Liang MZ Hu Q. Research progress of insomnia disorder at home and abroad. Med Philos B 2017;38:60–3.

[4] Janson C Lindberg E Gislason T Elmasry A Boman G. Insomnia in men – a 10-year prospective population based study. Sleep 2001;24:425–30. 11403527 10.1093/sleep/24.4.425

[5] Javaheri S Redline S. Insomnia and risk of cardiovascular disease. Chest 2017;152:435–44. 28153671 10.1016/j.chest.2017.01.026 PMC5577359

[6] Nishitani N Kawasaki Y Sakakibara H. Insomnia and depression: risk factors for development of depression in male Japanese workers during 2011-2013. Int J Public Health 2018;63:49–55. 29051985 10.1007/s00038-017-1043-9

[7] Islam MM Iqbal U Walther B. Benzodiazepine use and risk of dementia in the elderly population: a systematic review and meta-analysis. Neuroepidemiology 2016;47:181–91. 28013304 10.1159/000454881

[8] Pinto LR Jr Bittencourt LR Treptow EC Braga LR Tufik S. Eszopiclone versus zopiclone in the treatment of insomnia. Clinics (Sao Paulo, Brazil) 2016;71:05–9. 10.6061/clinics/2016(01)02 PMC4732384 26872077

[9] Roth T Seiden D Sainati S Wang-Weigand S Zhang J Zee P. Effects of ramelteon on patient-reported sleep latency in older adults with chronic insomnia. Sleep Med 2006;7:312–8. 16709464 10.1016/j.sleep.2006.01.003

[10] Gooneratne NS. Complementary and alternative medicine for sleep disturbances in older adults. Clin Geriatr Med 2008;24:121–38. 18035236 10.1016/j.cger.2007.08.002 PMC2276624

[11] Xue ZY. The world Federation of Chinese Medicine Societies Professional Committee of easy medicine umbilical needle was established in Beijing. Chin Acupunct Moxib 2016;36:1180.

[12] Xue ZY. The world Zhonglian Medical Umbilical Needle Professional Committee was established. World J Acupunct Moxib 2016;26:49.

[13] Dong ZH Qi Y. Umbilical acupuncture. Chin Acupunct Moxib 2002. 67–8.

[14] Yang W Zhang N Duan Q. Clinical application and research progress of umbilical needle therapy. Yunnan J Tradit Chin Med Mater Med 2018;39:88–90.

[15] Liu Y. Clinical observation of umbilical needle in treatment of insomnia. J Liaoning Univ Tradit Chin Med 2009;11:149–51.

[16] Dong ZH Qi Y. Clinical application of umbilical needle therapy. Shanghai J Tradit Chin Med 2004. 39–40.

[17] Man-hork L. Clinical Observation of Umbilical Acupuncture in the Treatment of Generalized Anxiety Disorder. Guangzhou: Traditional Chinese Medicine University of Guangzhou; 2019.

[18] Li GX Zhou CY Zhang XZ. Understanding of etiology and pathogenesis of insomnia in Traditional Chinese medicine. World J Sleep Med 2014;1:183–8.

[19] Qi Y. Umbilical needle therapy, umbilical holography and umbilical diagnosis. Chin Acupunct Moxib 2004. 70–5.

[20] Xiao HS Liu L. Based on brain–gut axis theory to explore the mechanism of gastric sedative therapy in the treatment of insomnia. J Chengdu Univ Chin Med 2021;44:108–13.

[21] Zhang LH Fang BW. Brain–gut axis and its role in the pathogenesis of gastrointestinal diseases. Chin J Integr Tradit West Med Surg 2007. 199–201.

[22] Luo Y Lv HT Ye H. Discuss the relationship between Bo's abdominal acupuncture therapy and Zang-fu meridian theory. J New Chin Med 2008. 104–5.

[23] Shamseer L Moher D Clarke M. Preferred reporting items for systematic review and meta-analysis protocols (PRISMA-P) 2015: elaboration and explanation. BMJ (Clin Res ed) 2015;350:g7647. 10.1136/bmj.g7647 25555855

[24] Harford TC Chen CM Saha TD Smith SM Hasin DS Grant BF. An item response theory analysis of DSM-IV diagnostic criteria for personality disorders: findings from the national epidemiologic survey on alcohol and related conditions. Personal Disord 2013;4:43–54. 22449066 10.1037/a0027416 PMC3760426

[25] Kupfer DJ Kuhl EA Wulsin L. Psychiatry's integration with medicine: the role of DSM-5. Annu Rev Med 2013;64:385–92. 23327527 10.1146/annurev-med-050911-161945

[26] Sateia MJ. International classification of sleep disorders-third edition: highlights and modifications. Chest 2014;146:1387–94. 25367475 10.1378/chest.14-0970

[27] Fung KW Xu J Bodenreider O. The new International Classification of Diseases 11th edition: a comparative analysis with ICD-10 and ICD-10-CM. J Am Med Inform Assoc JAMIA 2020;27:738–46. 32364236 10.1093/jamia/ocaa030 PMC7309235

[28] Chen YF. Chinese classification of mental disorders (CCMD-3): towards integration in international classification. Psychopathology 2002;35:171–5. 12145505 10.1159/000065140

[29] Buysse DJ Reynolds CF 3rd Monk TH Berman SR Kupfer DJ. The Pittsburgh Sleep Quality Index: a new instrument for psychiatric practice and research. Psychiatry Res 1989;28:193–213. 2748771 10.1016/0165-1781(89)90047-4

[30] Bastien CH Vallières A Morin CM. Validation of the Insomnia Severity Index as an outcome measure for insomnia research. Sleep Med 2001;2:297–307. 11438246 10.1016/s1389-9457(00)00065-4

[31] Liang MT Gao TS. Investigation and analysis of zang-fu syndrome in Guiding Principles for Clinical Research of New Chinese Medicine. Chin J Basic Med Tradit Chin Med 2008. 330–42.

[32] Higgins JP Altman DG Gøtzsche PC. The Cochrane Collaboration's tool for assessing risk of bias in randomised trials. BMJ (Clin Res ed) 2011;343:d5928. 10.1136/bmj.d5928 PMC3196245 22008217

[33] Higgins JP Thompson SG. Quantifying heterogeneity in a meta-analysis. Stat Med 2002;21:1539–58. 12111919 10.1002/sim.1186

[34] Balshem H Helfand M Schünemann HJ. GRADE guidelines: 3. Rating the quality of evidence. J Clin Epidemiol 2011;64:401–6. 21208779 10.1016/j.jclinepi.2010.07.015

[35] Chaput JP Yau J Rao DP Morin CM. Prevalence of insomnia for Canadians aged 6 to 79. Health Rep 2018;29:16–20. 30566205

